# TREM2 activation attenuates neuroinflammation and neuronal apoptosis via PI3K/Akt pathway after intracerebral hemorrhage in mice

**DOI:** 10.1186/s12974-020-01853-x

**Published:** 2020-05-28

**Authors:** Shengpan Chen, Jianhua Peng, Prativa Sherchan, Yongjie Ma, Sishi Xiang, Feng Yan, Hao Zhao, Yong Jiang, Ning Wang, John H. Zhang, Hongqi Zhang

**Affiliations:** 1 Department of Neurosurgery, Xuanwu Hospital, Capital Medical University, China International Neuroscience Institute (China-INI), No. 45 Changchun Street, Xicheng District, Beijing, 10053 China; 2 Department of Physiology and Pharmacology, Department of Neurosurgery and Anesthesiology, School of Medicine, Loma Linda University, Risley Hall, Room 219, 11041 Campus Street, Loma Linda, CA 92354 USA; 3 Department of Neurosurgery, The Affiliated Hospital of Southwest Medical University, Luzhou, 646000 China; 4 Laboratory of Neurological Diseases and Brain Functions, The Affiliated Hospital of Southwest Medical University, Luzhou, 646000 Sichuan China; 5 Department of Neurosurgery, Loma Linda University Medical Center, Loma Linda, CA 92354 USA

**Keywords:** Intracerebral hemorrhage, TREM2, ApoE, Neuroinflammation, Apoptosis

## Abstract

**Background:**

Neuroinflammation is an important host defense response to secondary brain injury after intracerebral hemorrhage (ICH). Triggering receptor expressed on myeloid cells 2 (TREM2) confers strong neuroprotective effects by attenuating neuroinflammation in experimental ischemic stroke. Recent studies suggest that apolipoprotein E (apoE) is a novel, high-affinity ligand of TREM2. This study aimed to investigate the effects of TREM2 activation on neuroinflammation and neuronal apoptosis in a mouse model of ICH.

**Methods:**

Adult male CD1 mice (*n* = 216) were subjected to intrastriatal injection of bacterial collagenase. The TREM2 ligand, apoE-mimetic peptide COG1410 was administered intranasally at 1 h after ICH induction. To elucidate the underlying mechanism, TREM2 small interfering RNA (siRNA) and the phosphatidylinositol 3-kinase (PI3K) inhibitor LY294002 were administered intracerebroventricularly prior to COG1410 treatment. Neurobehavioral tests, brain water content, immunofluorescence, western blotting, and Fluoro-Jade C- and terminal deoxynucleotidyl transferase dUTP nick end labeling staining were performed.

**Results:**

Endogenous TREM2 expression was increased and peaked at 24 h after ICH. TREM2 was expressed on microglia, astrocytes, and neurons. COG1410 improved both short-term and long-term neurological functions, reduced brain edema, inhibited microglia/macrophage activation and neutrophil infiltration, and suppressed neuronal apoptotic cell death in perihematomal areas after ICH. Knockdown of endogenous TREM2 by TREM2 siRNA aggravated neurological deficits and decreased the expression of TREM2 in naïve and ICH mice. COG1410 was associated with upregulation of TREM2, PI3K, phosphorylated-Akt, and Bcl-2 and downregulation of TNF-α, IL-1β, and Bax after ICH. The neuroprotective effects of COG1410 were abolished by both TREM2 siRNA and PI3K inhibitor LY294002.

**Conclusions:**

Our finding demonstrated that TREM2 activation improved neurological functions and attenuated neuroinflammation and neuronal apoptosis after ICH, which was, at least in part, mediated by activation of PI3K/Akt signaling pathway. Therefore, activation of TREM2 may be a potential therapeutic strategy for the management of ICH patients.

## Background

Intracerebral hemorrhage (ICH) is a common and severe cerebrovascular disease accounting for approximately 15 to 20% of all strokes, with high rates of mortality and morbidity [1]. The formation of hematoma and mechanical damage to adjacent tissues after sudden rupture of cerebral blood vessels results in a sharp increase in intracranial pressure, which is regarded as the primary brain injury [2]. However, surgical removal of the hematoma in clinical practice targeting primary brain injury after ICH shows no benefit to patients and rarely affects neurological recovery [3, 4]. Red blood cell debris and blood components trigger secondary brain injury following ICH, leading to an inflammatory response, oxidative stress, neuronal apoptosis, mitochondrial dysfunction, and blood-brain barrier (BBB) disruption [5, 6]. In recent decades, most experimental studies have focused on the mechanisms underlying ICH-induced secondary injury in search of novel therapeutic targets for ICH.

Neuroinflammation begins immediately after the formation of hematoma and is an important host defense response to secondary brain injury after ICH [7]. The inflammatory mechanisms include activation of microglia, the brain’s resident macrophages, and recruitment of peripheral leukocytes to the perihematomal region [5, 8]. Subsequently, activated microglia/macrophages and peripheral leukocytes release proinflammatory cytokines, including tumor necrosis factor (TNF)-α, interleukin (IL)-1β, chemokines, free radicals, and other toxic chemicals, which ultimately lead to brain edema, BBB disruption, and neuronal apoptosis [3, 5, 9]. ICH-induced neuronal apoptosis results in neutrophils and leukocytes infiltration into the brain, which further aggravates inflammatory injury [7, 10]. Therefore, anti-inflammatory treatment could provide a potential therapeutic strategy after the onset of ICH.

Triggering receptor expressed on myeloid cells 2 (TREM2) is an important innate immune receptor, which belongs to the lectin-like immunoglobulin superfamily and is primarily expressed on myeloid cells, such as immature dendritic cells, osteoclasts, tissue macrophages, and microglia [11, 12]. It contains an ectodomain, a transmembrane domain, and a short cytoplasmic tail [13]. TREM2 is implicated in a number of cellular processes including proliferation, survival, regulation of inflammatory cytokine production, and phagocytosis of apoptotic neuronal cells [14]. Although the precise endogenous ligand that activates TREM2 has yet to be fully characterized, recent studies suggest that apolipoprotein E (apoE) is a novel, high-affinity ligand of TREM2 [15–19]. Moreover, it has been demonstrated that overexpression of TREM2 could suppress inflammatory response in animal models of Alzheimer disease, Parkinson disease, multiple sclerosis, and other neurodegenerative diseases [20–22]. In recent years, accumulating evidence has shown that TREM2 confers strong neuroprotective effects by attenuating neuroinflammation in experimental ischemic stroke [23–26]. However, the potential neuroprotective role of TREM2 after ICH has yet to be elucidated.

As a lipid kinase, phosphatidylinositol 3-kinase (PI3K) can induce the phosphorylation of protein kinase B (Akt) to regulate cell survival, growth, and angiogenesis in response to extracellular signals [27]. Previous studies have demonstrated that PI3K/Akt signaling exerts anti-neuroinflammation, anti-oxidative stress, and anti-apoptotic properties in neurons [28, 29]. Interestingly, recent data revealed that PI3K/Akt signaling is the downstream target of TREM2 and is involved in TREM2-mediated inflammatory responses [27, 30–33].

In the present study, we hypothesized that activation of TREM2 with an apoE-mimetic peptide, COG1410, could improve neurological outcomes and attenuate neuroinflammation and neuronal apoptosis by modulating PI3K/Akt signaling pathway in a mouse model of ICH.

## Methods

### Animals

All experimental procedures were approved by the Institutional Animal Care and Use Committee of Loma Linda University and were performed according to the Guide for the Care and Use of Laboratory Animals of the National Institutes of Health and reported in compliance with the ARRIVE (Animal Research: Reporting of In Vivo Experiments) guidelines. A total of 216 male CD1 mice (8-week-old, weight 30–40 g; Charles River, Wilmington, MA, USA) were housed in a temperature and humidity controlled room under a standard 12-h light/dark cycle for a minimum of 3 days before ICH induction and provided free access to food and water.

### Experimental design

**Fig. 1:**
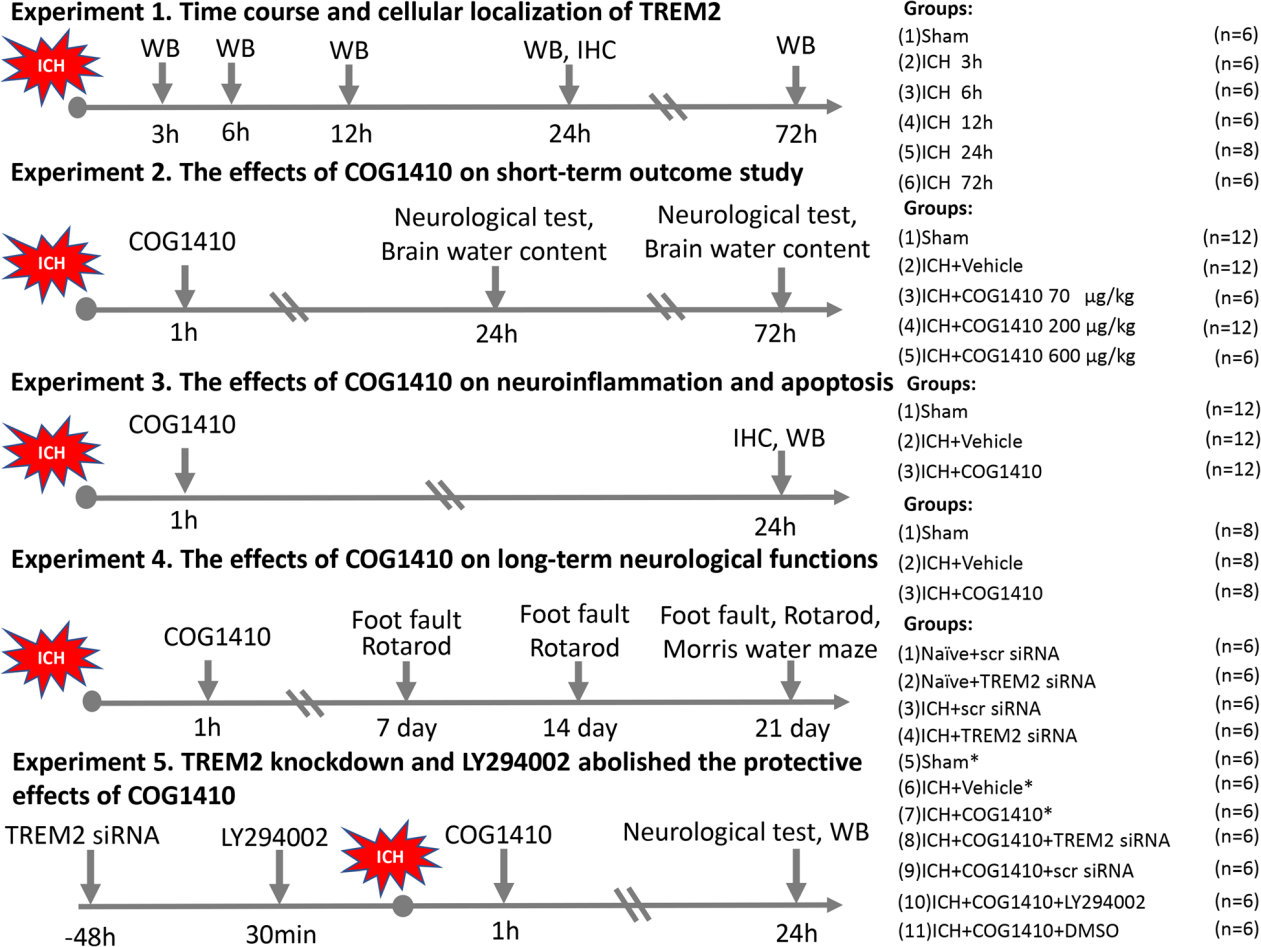
Experimental design and animal groups. ICH, intracerebral hemorrhage; WB, western blot; IHC, immunohistochemistry; DMSO, dimethyl sulfoxide. Asterisk indicates samples shared with experiment 3

In the current study, all mice were randomly assigned to the following five experimental procedures (Fig. 1) that were blinded to the researchers.

#### Experiment 1

To evaluate the time course of endogenous TREM2 expression in the ipsilateral/right hemisphere after ICH, 36 mice were randomly divided into six groups: sham, 3, 6, 12, 24, and 72 h after ICH (*n* = 6/group). Western blot analysis was performed to determine the changes in TREM2 expression. An additional two mice were used for double immunofluorescence staining at 24 h after ICH.

#### Experiment 2

To determine the effects of TREM2 activation with COG1410 on neuroinflammation, brain water content and neurobehavior tests were measured at 24 and 72 h after ICH. For effects of the drug at 24 h after ICH, 30 mice were assigned into five groups: sham, ICH + vehicle, ICH + COG1410 (70 μg/kg), ICH + COG1410 (200 μg/kg), and ICH + COG1410 (600 μg/kg) (*n* = 6/group). According to brain water content and neurobehavioral tests, a dosage of COG1410 at 200 μg/kg had the best treatment effect. For the effects of the drug at 72 h after ICH, 18 mice were randomly divided into three groups: sham, ICH + vehicle, and ICH + COG1410 (200 μg/kg) (*n* = 6/group).

#### Experiment 3

To assess the effects of COG1410 administration on microglia/macrophage activation, neutrophil infiltration, and neuronal cells death at 24 h after ICH, 18 mice were randomly divided into three groups for immunofluorescence, Fluoro-Jade C (FJC)-, and terminal deoxynucleotidyl transferase dUTP nick end labeling (TUNEL) staining: sham, ICH + vehicle, and ICH + COG1410 (200 μg/kg) (*n* = 6/groups). An additional 18 mice were randomly divided into three groups for western blot analysis: sham, ICH + vehicle, and ICH + COG1410 (200 μg/kg) (*n* = 6/groups).

#### Experiment 4

To investigate the effects of COG1410 on long-term neurobehavior functions after ICH, 24 mice were randomly assigned into three groups: sham, ICH + vehicle, and ICH + COG1410 (200 μg/kg) (*n* = 8/groups). The foot fault test and Rotarod test were performed on days 7, 14, and 21 after ICH. The Morris water maze was conducted on days 21–25 after ICH.

#### Experiment 5

To explore the underlying mechanisms of the neuroprotective role of COG1410, 42 mice were randomly divided into seven groups: sham, ICH + vehicle, ICH + COG1410 (200 μg/kg), ICH + COG1410 + TREM2 small interfering RNA (siRNA), ICH + COG1410 + scramble siRNA (scr siRNA), ICH + COG1410 + LY294002, and ICH + COG1410 + DMSO (*n* = 6/groups). The samples for sham, ICH + vehicle, and ICH + COG1410 groups were shared from experiment 3, and an additional 24 mice were used for the experiment. Additionally, to verify the efficacy of TREM2 siRNA to knockdown TREM2, 24 mice were randomly divided into four groups: naïve + scr siRNA, naïve + TREM2 siRNA, ICH + scr siRNA, and ICH + TREM2 siRNA (*n* = 6/groups). Neurobehavioral tests and western blot analysis were performed at 24 h after ICH.

### ICH model

Intracerebral hemorrhage was induced by stereotactic-guided injection of bacterial collagenase into the right basal ganglia as previously described [2]. Briefly, mice were anesthetized with a mixture of ketamine (100 mg/kg) and xylazine (10 mg/kg; 2:1, intraperitoneal injection) and positioned prone in a stereotaxic head frame (Kopf Instruments, Tujunga, CA, USA). An artificial tears ointment (Rugby, Livonia, MI, USA) was used to keep the eyes moist during surgery. A 1-mm cranial burr hole was drilled, and a 26-gauge needle of a 10-μL Hamilton syringe was inserted stereotactically into the right basal ganglia (coordinates 0.2 mm posterior, 2.2 mm lateral to bregma, and 3.5 mm below the dura). Bacterial collagenase type VII-S (0.075 units, Sigma-Aldrich, St. Louis, MO, USA) dissolved in 0.5 μL sterile phosphate-buffered saline (PBS) was infused into the brain at a rate of 0.167 μL/min with an infusion pump (Stoelting, Harvard Apparatus, Holliston, MA, USA). After injection, the needle was left in the position for an additional 5 min to prevent reflux and was withdrawn slowly at a rate of 1 mm/min. The cranial burr hole was sealed with bone wax, the scalp was sutured, and 0.4 mL of normal saline was injected subcutaneously to avoid postsurgical dehydration. Buprenorphine (0.03 mg/kg, Sigma-Aldrich) was administered subcutaneously to relieve the post-procedural pain. Mice were allowed to recover fully under close observation. The sham operation was performed with needle insertion only.

### Intranasal administration

Intranasal administration at 1 h after ICH was performed as previously described [34]. Saline or COG1410 at three different dosage (70 μg/kg, 200 μg/kg, and 600 μg/kg) dissolved in saline was administered intranasally. A total volume of 20 μL was delivered into the bilateral nares, alternating one naris at a time, with 5 μL per naris every 5 min for a period of 20 min.

### Intracerebroventricular injection

Intracerebroventricular administration was performed as previously described [2]. Briefly, a 26-gauge needle of a 10-μL Hamilton syringe was inserted into the left lateral ventricle through a cranial burr hole at the following coordinates relative to bregma: 0.3 mm posterior, 1.0 mm lateral, and 2.3 mm deep. A microinfusion pump was used for intracerebroventricular administration at a rate of 0.667 μL/min. The needle was left in place for an additional 8 min after the end of infusion and then removed over 3 min period. The burr hole was sealed with bone wax. The TREM2 siRNA (100 pmol/2 μl, OriGene Technologies, Rockville, MD, USA) or scr siRNA (100 pmol/2 μl, OriGene Technologies, Rockville, MD, USA) were delivered 48 h before ICH modeling. LY294002 (10 nmol/2 μl, Selleck Chemicals, Houston, TX, USA) or 25% DMSO (2 μl) was infused 30 min before ICH induction.

### Short-term neurobehavior assessment

Short-term neurological deficits were evaluated using the modified Garcia test, corner turn test, and forelimb placement test by two trained investigators blinded to experimental groups at 24 and 72 h after ICH, as previously described [35]. The modified Garcia test was assessed according to a 21-point score system with seven individual tests including spontaneous activity, axial sensation, vibrissae proprioception, limb symmetry, lateral turning, forelimb walking, and climbing. For the corner turn test, animals were allowed to advance into a 30° angle corner and exit by turning either to the left or right. Choice of turning was recorded for a total of ten trials, and a score was calculated as number of left turns/all trials × 100. For the forelimb placement test, the placement of ipsilateral forelimb on the countertop when the vibrissa was stimulated was recorded. The percent of the ipsilateral forelimb placement out of ten total trials was calculated.

### Brain water content measurement

Brain edema was evaluated by measuring brain water content using wet/dry method as previously described [36]. Briefly, mice were decapitated under deep anesthesia at 24 and 72 h after ICH, and the brains were immediately removed and divided into five parts: the ipsilateral and contralateral cortices, the ipsilateral and contralateral basal ganglia, and the cerebellum. Each part was immediately weighed on an electric analytic balance (APX-60, Denver Instrument, Bohemia, NY, USA) to obtain the wet weight and then dried for 24 h at 100 °C to obtain the dry weight. Brain water content was calculated using the following formula: brain water content (%) = [(wet weight − dry weight)/wet weight] × 100%.

### Long-term neurobehavior assessment

The foot fault test and Rotarod test were performed to assess sensorimotor coordination and balance in the first, second, and third week after ICH, whereas the Morris water maze was performed to evaluate spatial learning and memory abilities on days 21 to 25 after ICH, as previously reported [34]. Briefly, for the foot fault tests, mice were allowed to move along a horizontal wire grid (20 × 100 cm) for 2 min and the number of left forelimb missteps was recorded. For the Rotarod test, the rotating speed was started at 5 revolutions/min and gradually accelerated by 2 revolutions/min every 5 s. The duration that mice were able to stay on the accelerating rotating cylinder was recorded by a photobeam circuit. For the Morris water maze, mice were placed in a semi-random set of start locations to find a visible platform above the water level in 60 s. After, the mice were guided to stay on the platform for 5 s and their swim path, escape latency, and swim distance were recorded individually. On the last day, the probe trial was performed in which the platform was removed and the duration of time spent in the probe quadrant was recorded. A computerized tracking system (Noldus Ethovision, Tacoma, WA, USA) was used to record all data.

### Western blotting analysis

Western blotting was performed as previously described [6]. Briefly, after brain protein sample preparation using RIPA lysis buffer (Santa Cruz Biotechnology, Santa Cruz, CA, USA), equal amounts of protein were loaded on an SDS-PAGE gel and run using electrophoresis and then transferred to a nitrocellulose membrane. The membrane was blocked and incubated overnight at 4 °C with the following primary antibodies: goat anti-TREM2 (1:1000, Abcam, Cambridge, MA, USA), rabbit anti-PI3K (1:1000, Cell signaling, Danvers, MA, USA), rabbit anti-phosphorylated Akt (p-Akt, 1:1000, Cell signaling), rabbit anti-Akt (1:1000, Cell signaling), rabbit anti-TNF-α (1:1000, Abcam), rabbit anti-IL-1β (1:1000, Abcam), anti-Bcl-2 (1:2000, Abcam), anti-Bax (1:4000, Abcam), and goat anti-β-actin (1:5000, Santa Cruz Biotechnology). Appropriate secondary antibodies (1:3000, Santa Cruz; 1:5000, Abcam) were selected to incubate with the membrane for 2 h at room temperature. The bands were probed with an ECL Plus chemiluminescence regent Kit (Amersham Biosciences, Arlington Heights, PA, USA) and visualized with the image system (Versa Doc, model 4000, Bio-Rad, Hercules, CA, USA). Relative density of the protein immunoblot images were analyzed by ImageJ software (ImageJ 1.4, NIH, Bethesda, MD, USA).

### Immunofluorescence staining

Briefly, after mice were anesthetized with isoflurane and intracardially perfused with ice-cold PBS and 10% formalin, the brains were removed, fixed in 10% formalin overnight at 4 °C, and dehydrated with 30% sucrose for 3 days. Brain tissues were snap-frozen at − 80 °C and cut into 10-μm-thick coronal sections using a cryostat (CM3050S; Leica Microsystems, Bannockburn, III, Germany). Immunofluorescence staining was performed as previously described [7]. Brain samples were incubated overnight at 4 °C with primary antibodies including rabbit anti-ionized calcium-binding adaptor molecule 1 (Iba-1, 1:100, Abcam), rabbit anti-glial fibrillary acidic protein (GFAP, 1:100, Abcam), rabbit anti-neuronal specific nuclear protein (NeuN, 1:200, Abcam), rabbit anti-myeloperoxidase (MPO, 1:500, Abcam), and goat anti-TREM2 (1:1000, Abcam). The sections were then incubated with corresponding secondary antibodies (1:200, Jackson Immunoresearch, West Grove, PA, USA) for 2 h at room temperature, followed by visualization using a fluorescence microscope (Leica Microsystems).

### FJC staining

Degenerating neurons were evaluated by FJC staining as previously reported [37]. According to manufacturer’s instructions, slides were immersed in 1% sodium hydroxide solution for 5 min, followed by rinsing in 70% ethanol for 2 min, and were subsequently transferred into distilled water for 2 min. After being incubated in 0.06% potassium permanganate solution for 10 min, the sliders were rinsed in distilled water for 2 min and transferred into a 0.0001% solution of FJC (Millipore, Billerica, MA, USA) dissolved in 0.1% acetic acid for 10 min. Slides were rinsed with distilled water three times for 1 min each, dried in a slide incubator for 5 min at 50 °C, immersed in xylene for 1 min, and finally cover slipped with DPX (Sigma-Aldrich). The sections were visualized in blinded manner with a fluorescence microscope, Leica DMi8 (Leica Micro-systems). FJC-positive neurons were manually counted in the peri-hematoma regions of six sections per brain at × 200 magnification using ImageJ software (Image J 1.4, NIH). The data were averaged and expressed as positive cells/mm^2^.

### TUNEL staining

For quantification of neuronal apoptosis, double staining of NeuN (green) and TUNEL (red) was performed using in situ Apoptosis Detection Kit (Roche, Indianapolis, IN, USA) according to the manufacturer’s instructions at 24 h after ICH [38]. The number of TUNEL-positive neurons was counted manually in the peri-hematoma area of six sections per brain at × 200 magnification using Image J software (Image J 1.4, NIH). Data was expressed as the ratio of TUNEL-positive neurons (%).

### Statistical analysis

All data were expressed as the mean and standard deviation (mean ± SD). Statistical analysis was performed with Graph Pad Prism (Graph Pad Software, San Diego, CA, USA). Statistical evaluation of the data was performed by analysis of variance (ANOVA), followed by Tukey multiple-comparison post hoc analysis. Sample sizes were calculated assuming a type I error rate = 0.05 and power = 0.8 on a two-sided test in this study. Statistical significance was defined as *p* < 0.05.

## Results

### Animal mortality and exclusion

Of a total of 216 mice used, 166 mice were subjected to ICH. No mice died in sham or naïve groups. The overall mortality of mice was 10.84% (18/166). Four mice were excluded from this study due to lack of hemorrhage. The summary of experimental groups, animal numbers, and mortality rate are listed in a table (Additional file 1: Table S1).

### Time course and spatial expressions of TREM2 after ICH

**Fig. 2:**
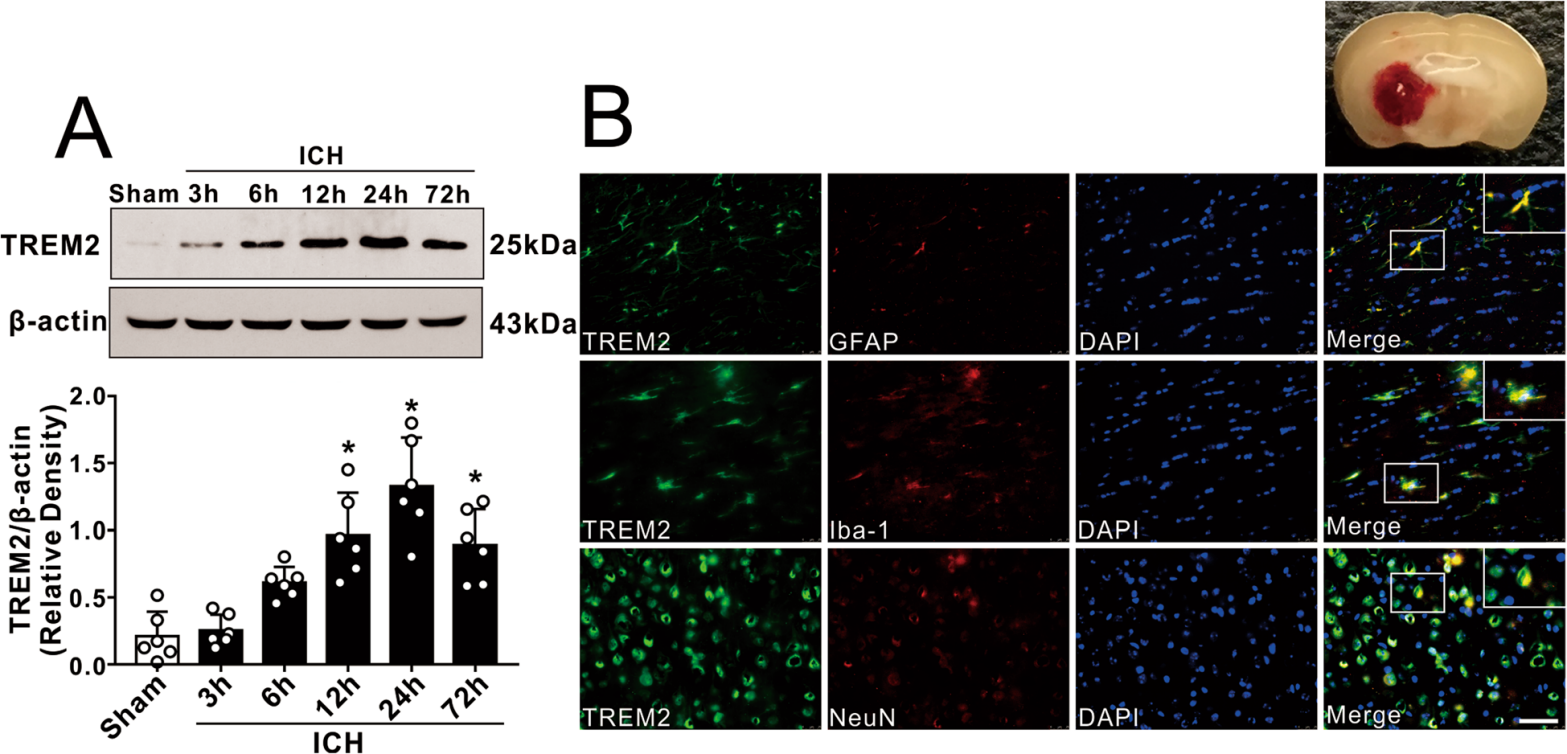
Expression profile of TREM2 after ICH. **a** Representative western blot bands of time course and quantitative analyses of TREM2 expression in the ipsilateral hemisphere after ICH. *p < 0.05 vs. sham. Error bars are represented as mean ± SD. *n* = 6 per group. **b** Representative images of colocalization of TREM2 (green) with astrocytes (GFAP, red), microglia/macrophage (Iba-1, red), and neurons (NeuN, red) in the perihematomal area at 24 h after ICH. Nuclei were stained with DAPI (blue). Scale bar = 50 μm, *n* = 2

The endogenous expression of TREM2 was assessed by western blot analysis at 0 (sham), 3, 6, 12, 24, and 72 h in the ipsilateral/right cerebral hemispheres after ICH. The results showed that the expression of TREM2 was significantly increased at 12 h, reached to the highest level at 24 h, and decreased at 72 h after ICH when compared with sham group (*p* < 0.05, Fig. 2a). Double immunofluorescence staining was performed to determine the cellular localization of TREM2 at 24 h after ICH. The results showed that TREM2 was expressed in microglia (Iba-1), astrocytes (GFAP), and neurons (NeuN) in the perihematomal area at 24 h after ICH (Fig. 2b).

### COG1410 treatment reduced brain edema and attenuated neurological deficits at 24 and 72 h after ICH

**Fig. 3:**
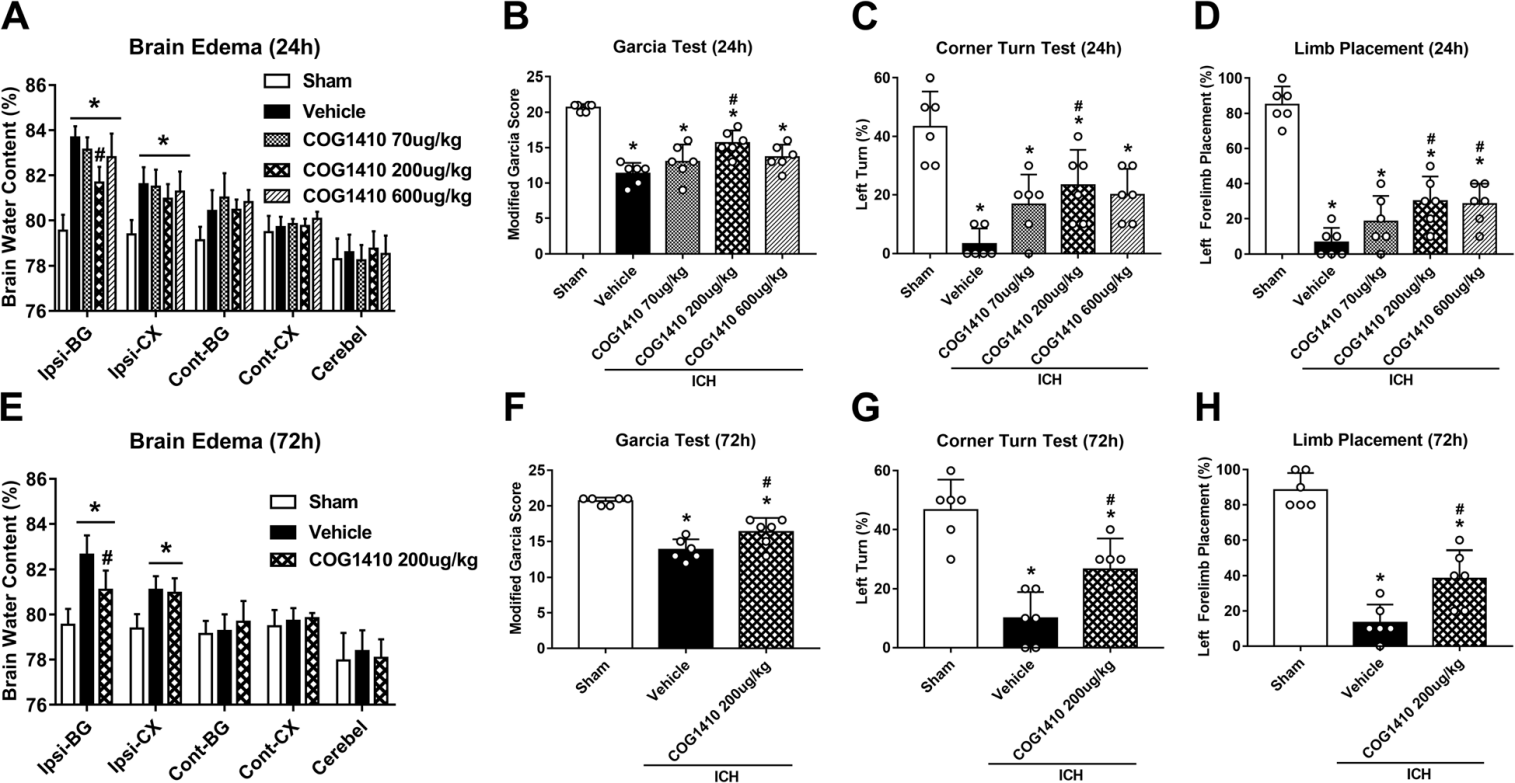
The effects of COG1410 on neurobehavioral outcomes and brain water content after ICH. **a** Brain water content, **b** modified Garcia test, **c** corner turn test, and **d** forelimb placement test at 24 h after ICH. **e** Brain water content, **f** modified Garcia test, **g** corner turn test, and **h** forelimb placement test at 72 h after ICH. *p < 0.05 vs. sham, ^#^*p* < 0.05 vs. ICH + vehicle. Error bars are represented as mean ± SD. *n* = 6 per group. Ipsi-BG, ipsilateral basal ganglia; Ipsi-CX, ipsilateral cortex; Cont-BG, contralateral basal ganglia; Cont-CX, contralateral cortex; Cerebel, cerebellum

Three different dosages of COG1410 were used to choose the optimal dosage in attenuating ICH-induced brain injury. The brain water content in the ipsilateral basal ganglia and ipsilateral cortex was significantly increased in the ICH groups when compared with sham group at 24 h after ICH (*p* < 0.05, Fig. 3a, e), which was significantly reduced after COG1410 administration (200 μg/kg) (*p* < 0.05, Fig. 2a, e). Significant neurological deficits were observed in ICH groups at 24 h when compared with sham group at 24 h as assessed by the modified Garcia test (*p* < 0.05, Fig. 3b), corner turn test (*p* < 0.05, Fig. 3c), and forelimb placement test (*p* < 0.05, Fig. 3d). Administration of COG1410 (200 μg/kg) significantly improved neurological outcomes (*p* < 0.05, Fig. 3b–d) at 24 h after ICH when compared with ICH + vehicle group. To further verify the treatment efficacy of COG1410 (200 μg/kg), brain water content and neurobehavioral tests were also performed at 72 h after ICH. Consistently, ICH + COG1410 (200 μg/kg) group significantly reduced brain water content in the ipsilateral basal ganglia and ipsilateral cortex (*p* < 0.05, Fig. 3e) and improved neurological functions (*p* < 0.05, Fig. 3f–h) when compared with ICH + vehicle group at 72 h after ICH. Therefore, a middle dosage of COG1410 was selected for long-term and mechanistic studies.

### COG1410 treatment inhibited microglia/macrophage activation, neutrophil infiltration, and the expression of TNF-α and IL-1β at 24 h after ICH

**Fig. 4:**
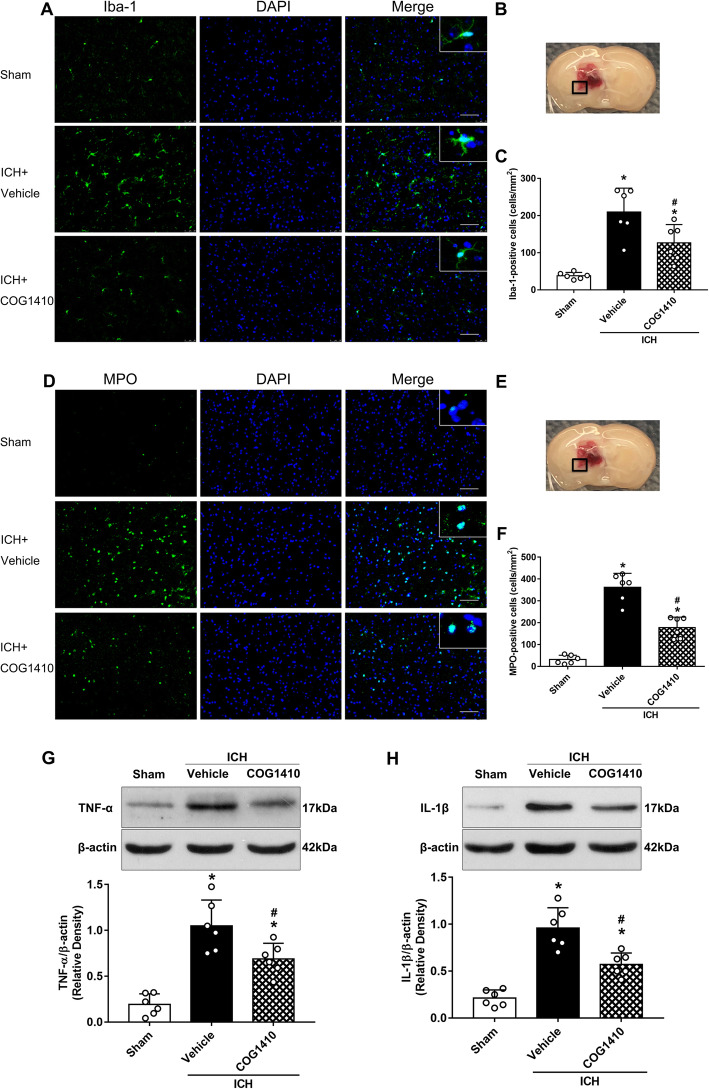
The effects of COG1410 on microglia/macrophage activation and neutrophil infiltration after ICH. **a**, **d** Representative images of immunofluorescence staining of Iba-1 (green) and MPO (green) in the perihematomal area at 24 h after ICH. **b**, **e** Brain sample with schematic illustration showing the area (indicated by black squares) used for Iba-1 and MPO-positive cell counting in the perihematomal region. **c**, **f** Quantitative analyses of Iba-1 and MPO-positive cells in the perihematomal area at 24 h after ICH. **g**, **h** Representative western blot bands and quantitative analyses of TNF-α and IL-1β protein levels in the ipsilateral hemisphere at 24 h after ICH. *p < 0.05 vs. sham, ^#^*p* < 0.05 vs. ICH + vehicle. Error bars are represented as mean ± SD. *n* = 6 per group

The levels of Iba-1 and MPO in the perihematomal area at 24 h after ICH were performed to detect microglia/macrophage activation and neutrophil infiltration by immunofluorescence staining. The results showed that the numbers of Iba-1 and MPO-positive cells in the perihematomal area were significantly increased in ICH + vehicle group when compared with sham group at 24 h after ICH (*p* < 0.05, Fig. 4a–f). However, COG1410 treatment significantly decreased the numbers of Iba-1 and MPO-positive cells in the perihematomal area when compared with ICH + vehicle group at 24 h after ICH (*p* < 0.05, Fig. 4a–f). Additionally, the expression of proinflammatory mediators TNF-α and IL-1β at 24 h after ICH were measured by western blot. The results showed that the expression of TNF-α and IL-1β in the ipsilateral hemisphere were significantly decreased with COG1410 treatment when compared with ICH + vehicle group at 24 h after ICH (*p* < 0.05, Fig. 4g, h).

### COG1410 treatment attenuated neuronal apoptotic death and the expression of Bcl-2 and Bax at 24 h after ICH

**Fig. 5:**
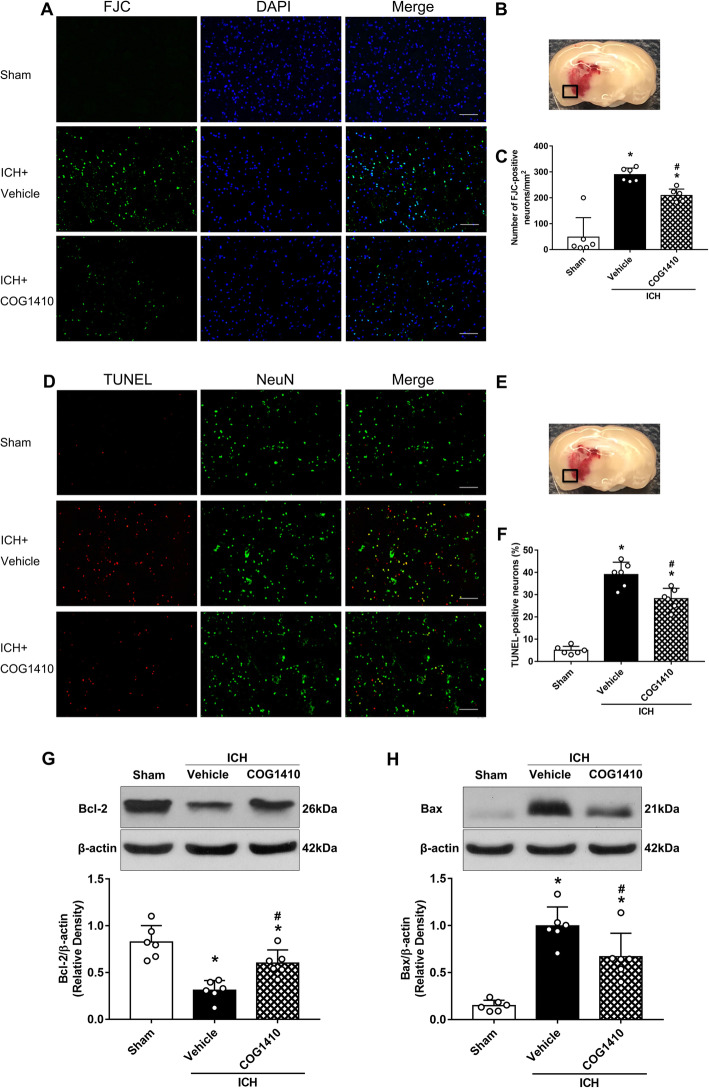
The effects of COG1410 on neuronal apoptosis and proinflammatory factors after ICH. **a**, **d** Representative images of FJC (green), and the colocalization of TUNEL (red) with neurons (NeuN, green) in the perihematomal area at 24 h after ICH. **b**, **e** Brain sample with schematic illustration showing the area (indicated by black squares) used for FJC and TUNEL counting in the perihematomal region. **c**, **f** Quantitative analyses of FJC and TUNEL-positive cells in the perihematomal area at 24 h after ICH. **g**, **h** Representative western blot bands and quantitative analyses of TNF-α and IL-1β protein levels in the ipsilateral hemisphere at 24 h after ICH. *p < 0.05 vs. sham, ^#^*p* < 0.05 vs. ICH + vehicle. Error bars are represented as mean ± SD. *n* = 6 per group

Degenerating and apoptotic neurons in the perihematomal area at 24 h after ICH were assessed by FJC and TUNEL staining. The results revealed that FJC-positive neurons and TUNEL-positive neurons of the perihematomal area in ICH + vehicle group significantly increased when compared with sham group at 24 h after ICH, but that COG1410 treatment reduced the numbers of FJC-positive neurons and TUNEL-positive neurons (*p* < 0.05, Fig. 5a–f). Meanwhile, the expression of apoptotic molecular markers Bcl-2 and Bax at 24 h after ICH were measured by western blot. The results showed that the expression of Bcl-2 was significantly increased and the expression of Bax was significantly decreased with COG1410 treatment when compared with ICH + vehicle group in the ipsilateral hemisphere at 24 h after ICH (*p* < 0.05, Fig. 5g, h).

### COG1410 treatment improved long-term neurological functions after ICH

**Fig. 6:**
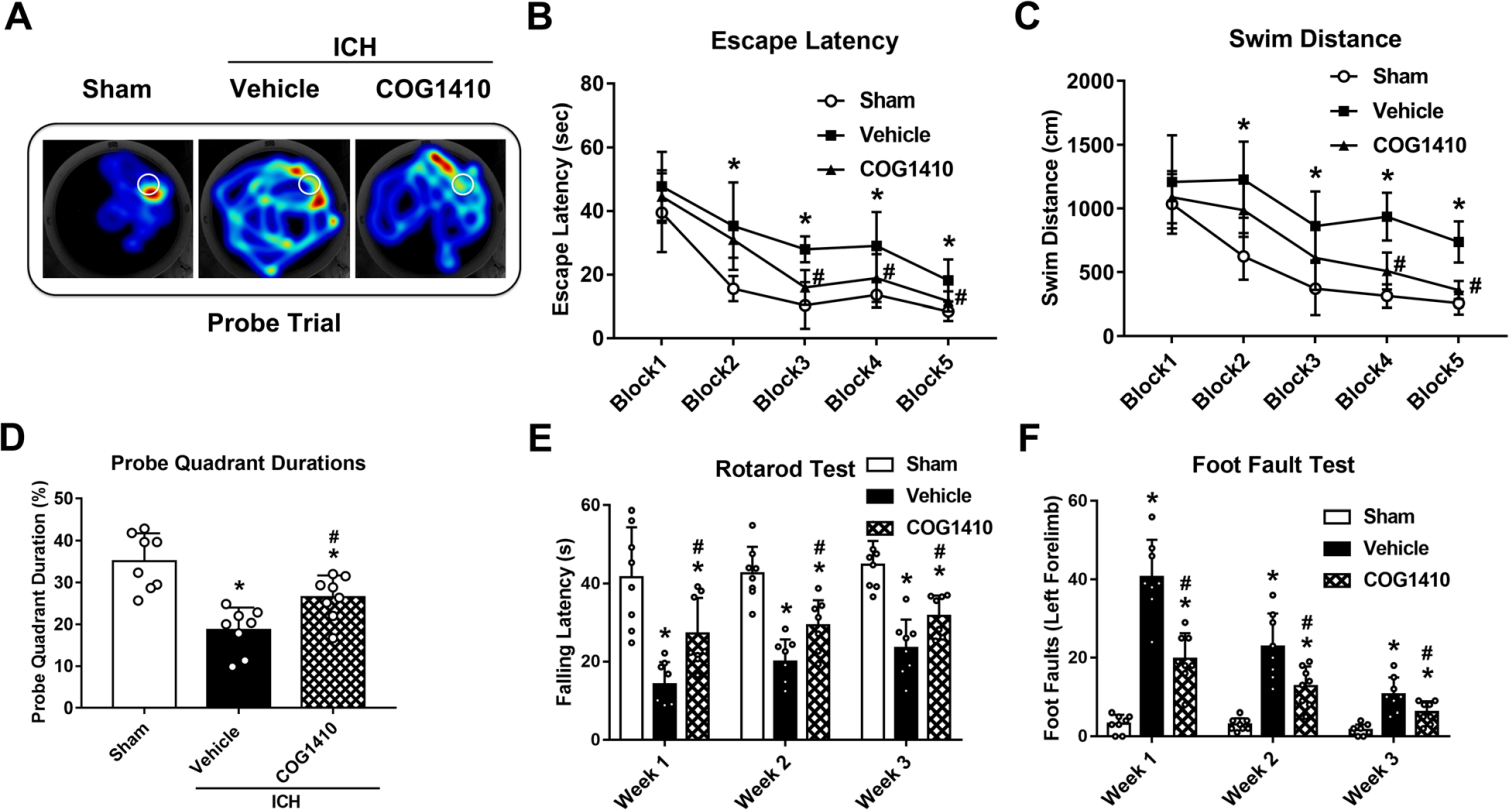
The effects of COG1410 on long-term neurobehavioral outcomes after ICH. **a** Typical traces of Morris water maze on day 25 after ICH. **b**, **c** Escape latency and swim distance of Morris water maze on days 21 to 25 after ICH. **d** Probe quadrant duration of Morris water maze on day 25 after ICH. **e**, **f** Rotarod test and Foot fault test and in the first, second, and third week after ICH. *p < 0.05 vs. sham, ^#^*p* < 0.05 vs. ICH + vehicle. Error bars are represented as mean ± SD. *n* = 8 per group

In the probe quadrant trial, the mice in ICH + vehicle group spent remarkably less time in the target quadrant when compared with sham group, while the reference memory deficits were significantly increased with COG1410 treatment (*p* < 0.05, Fig. 6a, d). In the Morris water maze test, escape latency and swim distance to find the platform were significantly increased in ICH + vehicle group when compared with sham group (*p* < 0.05, Fig. 6b, c). However, a significant decrease in escape latency on blocks 3 to 5 and a significantly shorter swim distance on blocks 4 and 5 were observed in ICH + COG1410 group (*p* < 0.05, Fig. 6b, c).

In the Rotarod test and foot fault tests, the mice in ICH + vehicle group had significantly shorter falling latency and more foot faults of the left forelimb when compared with sham groups in the first, second, and third week after ICH (*p* < 0.05, Fig. 6e, f). However, COG1410 treatment significantly improved the neurological deficits when compared with the ICH + vehicle group (*p* < 0.05, Fig. 6e, f).

### TREM2 siRNA aggravated neurological deficits and decreased the expression of TREM2 in naïve and ICH mice

**Fig. 7:**
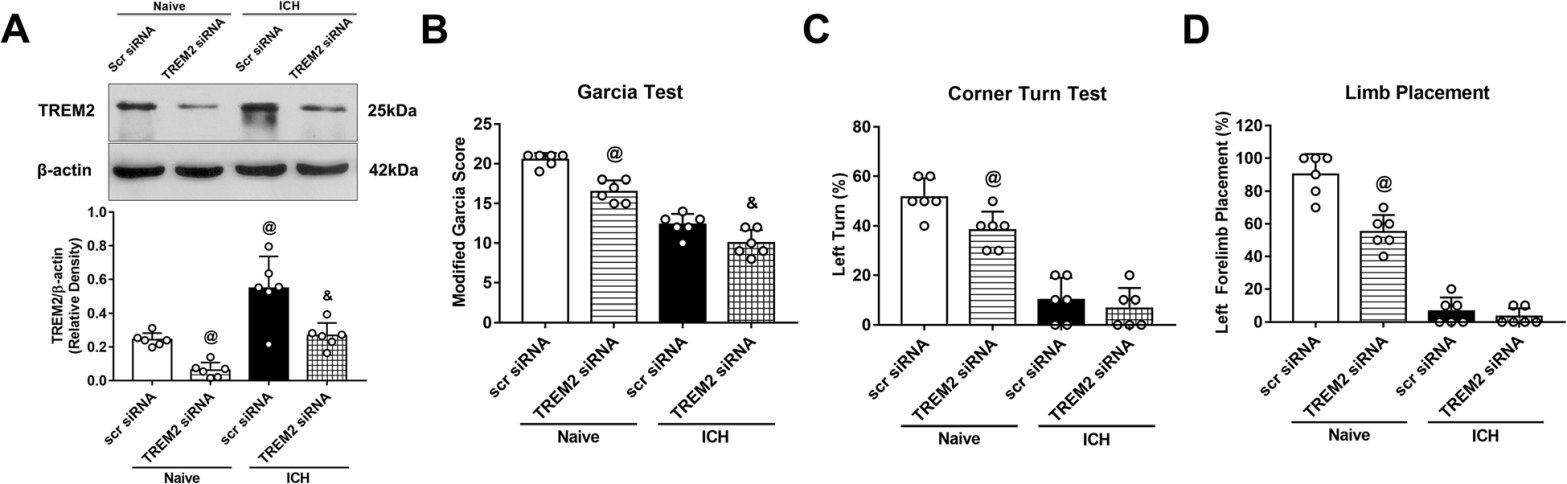
TREM2 deletion aggravated neurological deficits. **a** Representative western blot bands and quantitative analyses of TREM2 protein levels in the ipsilateral hemisphere (right) of naïve mice or ICH mice. **b**–**d** The modified Garcia test, corner turn test, and forelimb placement test of each group. ^@^*p* < 0.05 vs. naïve + scr siRNA, ^&^*p* < 0.05 vs. ICH + scr siRNA. Error bars are represented as mean ± SD. *n* = 6 per group

The knockdown efficacy of TREM2 siRNA was verified by western blotting and neurobehavior tests in naïve and ICH mice. TREM2 siRNA administered by intracerebroventricular injection significantly decreased the expression of TREM2 in the ipsilateral hemisphere and aggravated neurological deficits in naïve + TREM2 siRNA group when compared with naïve+ scr siRNA group (*p* < 0.05, Fig. 7a–d). Similarly, the expression of TREM2 was significantly decreased and neurological deficits was aggravated in ICH + TREM2 siRNA group when compared with ICH + scr siRNA group (*p* < 0.05, Fig. 7a–d).

### TREM2 siRNA and LY294002 abolished the neuroprotective effects of COG1410 on neurological functions at 24 h after ICH

**Fig. 8:**
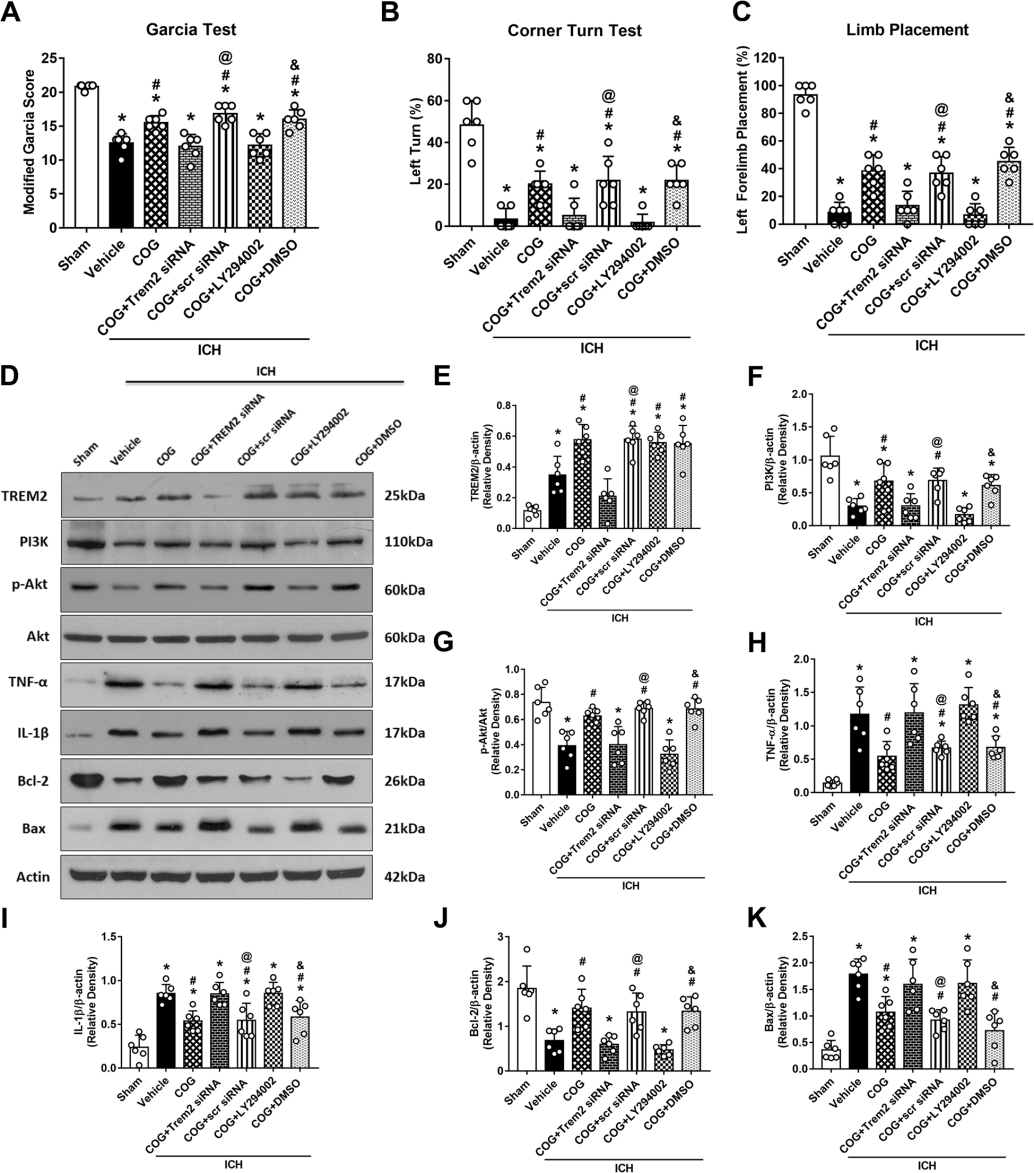
TREM2 knockdown and PI3K inhibition reversed the effects of COG1410 on neurological functions and inflammatory/apoptotic proteins expression. **a**–**c** The modified Garcia test, corner turn, test and forelimb placement test of each group at 24 h after ICH. **d** Representative western blot bands. **e**–**k** Quantitative analyses of TREM2, PI3K, phosphorylated Akt, TNF-α, IL-1β, Bcl-2, and Bax in the ipsilateral hemisphere at 24 h after ICH. *p < 0.05 vs. sham, ^#^*p* < 0.05 vs. ICH + vehicle, ^@^*p* < 0.05 vs. ICH + COG1410 + TREM2 siRNA, ^&^*p* < 0.05 vs. ICH + COG1410 + LY294002. Error bars are represented as mean ± SD. *n* = 6 per group

Administration of TREM2 siRNA significantly aggravated the neurological deficits assessed by the modified Garcia (*p* < 0.05, Fig. 8a), corner turn (*p* < 0.05, Fig. 8b), and forelimb placement tests (*p* < 0.05, Fig. 8c) in ICH + COG1410 + TREM2 siRNA group when compared with ICH + COG1410 + scr siRNA group at 24 h after ICH. Similarly, LY294002 treatment exacerbated the neurological deficits in ICH + COG1410 + LY294002 group when compared with ICH + COG1410 + DMSO group at 24 h after ICH (*p* < 0.05, Fig. 8a–c).

### COG1410 suppressed neuroinflammation and neuronal apoptosis via activation of the TREM2/PI3K/Akt signaling pathway at 24 h after ICH

After COG1410 treatment, the expression of TREM2 significantly increased when compared with both sham and ICH + vehicle groups (*p* < 0.05, Fig. 8d, e). Moreover, the expression of PI3K, p-Akt, and Bcl-2 was significantly decreased, while the expression of TNF-α, IL-1β, and Bax was remarkably increased in ICH + vehicle group when compared with sham group at 24 h after ICH (*p* < 0.05, Fig. 8d, f–k). COG1410 treatment significantly increased the expression of PI3K, p-Akt, and Bcl-2 and significantly decreased the expression of TNF-α, IL-1β, and Bax in ICH + COG1410 group when compared with ICH + vehicle group at 24 h after ICH (*p* < 0.05, Fig. 8d, f–k). However, knockdown of TREM2 with TREM2 siRNA remarkably decreased the expression of TREM2, PI3K, p-Akt, and Bcl-2 and increased the expression of TNF-α, IL-1β, and Bax in ICH + COG1410 + TREM2 siRNA group when compared with ICH + COG1410 + scr siRNA group at 24 h after ICH (*p* < 0.05, Fig. 8d–k). Consistently, pretreatment with LY294002 significantly decreased the expression of PI3K, p-Akt, and Bcl-2, while increased the expression of TNF-α, IL-1β, and Bax in ICH + COG1410 + LY294002 group when compared with ICH + COG1410 + DMSO group at 24 h after ICH (*p* < 0.05, Fig. 8d, f–k).

## Discussion

In the current study, we first investigated the neuroprotective effects of TREM2 activation and explored its underlying mechanism in a mouse model of ICH. We demonstrated that activation of TREM2 with COG1410 improved both short- and long-term neurological functions, reduced brain edema, inhibited microglia/macrophage activation and neutrophil infiltration, and suppressed neuronal apoptotic cell death in perihematomal areas after ICH. In addition, knockdown of endogenous TREM2 by TREM2 siRNA aggravated neurological deficits and decreased the expression of TREM2 in naïve and ICH mice. Moreover, intranasal administration of COG1410 was associated with upregulation of TREM2, PI3K, p-Akt, and Bcl-2 and with downregulation of TNF-α, IL-1β, and Bax after ICH. However, blockage of TREM2 or silencing of PI3K reversed the beneficial effects of COG1410 on neurological functions and the expression of proinflammatory mediators and apoptotic markers. Finally, our findings suggested that TREM2 activation may attenuate neuroinflammation and neuronal apoptosis after ICH, which was, at least in part, mediated by activation of PI3K/Akt signaling pathway.

There are two major and best-characterized members in the TREM family, TREM1 and TREM2, which are immunomodulatory receptors that have important roles in innate and adaptive immunity. TREM1 is thought to be an amplifier of the immune response, while TREM2 is believed to be a negative regulator [39]. TREM2 protein is mainly expressed on myeloid lineage cells and, in particular, on microglial cells, which have approximately 300-fold higher densities of the protein than neurons and other glial cells in the central nervous system (CNS) [40, 41]. Interestingly, our immunofluorescence staining results showed that TREM2 was expressed not only on microglia but also on astrocytes and neurons following ICH. We also observed that the endogenous expression of TREM2 in the ipsilateral/right hemisphere of brain was increased in a time-dependent manner and reached a peak at 24 h after ICH. A previous study reported that the expression of TREM2 was upregulated as early as 6 h and peaked at approximately 72 h in a rat model of cerebral ischemia [23]. Another study demonstrated that mRNA and protein expression of TREM2 were both markedly increased in the rat brain after ischemia/reperfusion injury [25]. Our data were consistent with previous studies, but the mechanisms of the TREM2 change after ICH remain incompletely understood. It is well known that microglia activation occurs as early as 1 h in a collagenase-induced ICH model and that the number of activated microglia/macrophages peaks at 72 h and returns to normal level several weeks after the insult [42]. Moreover, TREM2 is primarily expressed on microglia in the brain. Given the above, TREM2 may be upregulated to regulate the inflammatory response during the acute phase of ICH.

Numerous studies have revealed that TREM2 exerts anti-inflammatory properties and promotes phagocytosis of apoptotic neuronal cells in neurodegenerative diseases, liver ischemia/reperfusion injury, and bacterial infections [20, 21, 32, 43, 44]. A previous study reported that TREM2 deficiency exacerbated uncontrolled inflammation in a model of autoimmune encephalomyelitis [45]. Increasing evidence has shown that upregulation of TREM2 results in strong neuroprotective effects by alleviating neuroinflammation in experimental cerebral ischemia [24, 25, 33]. Specifically, an in vivo and in vitro study demonstrated that TREM2 activation promoted microglial switching from the detrimental M1 phenotype to the beneficial M2 phenotype and that as a result decreased the number of apoptotic neurons after ischemic damage [23]. However, the potential therapeutic effects targeting TREM2 in hemorrhagic stroke have yet to be elucidated.

Recent studies identified that apoE is a novel and high-affinity ligand for TREM2 and that the TREM2-binding domain is found in amino acids 130-149 of apoE [15–19]. ApoE is the major apolipoprotein synthesized in the CNS, which not only serves as an important mediator of cholesterol and lipid transport, but also affords neuroprotective effects via multiple mechanisms including anti-inflammatory, anti-excitotoxic, and anti-oxidant properties after brain injury [46–48]. ApoE exists three isoforms, designated apoE2, apoE3, and apoE4. However, apoE4 is associated with higher risk of cardiovascular morbidity, cognitive impairments during aging, and Alzheimer’s disease [49, 50]. However, like most other proteins, the intact apoE holoprotein is too large to cross the BBB because of its 34-kDa molecular weight. Thus, from a translational perspective, the development of apoE-mimetic peptides with smaller molecular weights as alternative therapeutic choices for CNS disease treatment is greatly desirable.

COG1410 is a peptide derived from apoE amino acid residues 138–149 of the receptor region of the apoE holoprotein with aminoisobutyric acid (Aib) substitutions at positions 140 and 145 (acetyl-AS-Aib-LRKL-Aib-KRLL-amide). Emerging studies have indicated that brain edema is significantly associated with hematoma enlargement and increased midline shift, which lead to poor neurological outcome after ICH [51]. In our study, we found that COG1410 treatment reduced brain edema and attenuated neurological deficits at 24 and 72 h after ICH. Furthermore, COG1410 treatment improved long-term movement coordination ability, spatial learning, and memory. In addition, ICH-induced microglia/macrophage activation and neutrophil infiltration can further promote the release of proinflammatory mediators and neuronal apoptotic molecular markers, which ultimately result in neuroinflammation and neuronal apoptosis [5]. On the contrary, several studies have demonstrated that COG1410 treatment improves functional recovery and alleviates neuroinflammation and neuronal apoptosis in experimental ischemic stroke, hemorrhage stroke, and traumatic brain injury [52–55].

Consistent with previous studies, our data showed that COG1410 treatment inhibited microglia/macrophage activation, neutrophil infiltration, and neuronal apoptotic death as measured by immunofluorescence, and FJC and TUNEL staining, and downregulated the expression of TNF-α, IL-1β, Bcl-2 and Bax, as measured by western blotting, at 24 h after ICH. Furthermore, our data showed that TREM2 knockdown by siRNA and PI3K inhibition by the specific inhibitor LY294002 significantly reversed the anti-inflammatory and anti-apoptotic effects of apoE-mimic peptide COG1410. These data suggested the apoE-mimic peptide inhibited neuroinflammation and neuronal apoptosis by activating the PI3K/Akt pathway through TREM2 after ICH.

Several limitations need to be mentioned in this study. First, TREM2 plays multifunctional roles in innate and adaptive immunity. Further research is needed to investigate the other mechanisms underlying the neuroprotective effects of TREM2 against secondary brain injury after ICH. Second, since our double immunofluorescence staining showed that TREM2 is not only expressed on microglia and neurons after ICH, detailed roles of TREM2 on other CNS cells, such as astrocytes, are necessary for further study. Third, apoE has been shown to be bound and internalized via receptor-mediated endocytosis by other receptors, including the low-density lipoprotein receptor, very-low density lipoprotein receptor, and apoE receptor 2 in the CNS. How the apoE-mimic peptide affects these receptors and the related pathways and the specific mechanism associated with peptide activation of TREM2 should be more deeply evaluated. Besides, the silencing efficiency of TREM2 siRNA upon local injection in mouse brain is also a concern in our research process. Recent studies and our present study have demonstrated effectiveness of local knockdown of genes using siRNAs in the brain [56, 57]. However, the time window and tissue area of siRNA are very limited, because of the poor intracellular uptake and low blood stability of siRNA. This study only focused on the early pathophysiological changes (within 72 h) around the hematoma after ICH, the effects of TREM2 on specific regions/cell types, and the long-term prognosis after ICH require more precise gene-editing techniques, such as the use of cre/loxp mice to edit TREM2 genes in specific cells. Last but not least, since bacterial collagenase was used to induce ICH in this study, we cannot exclude the possibility of initiating greater neuroinflammation than that found in autologous blood-induced ICH model. Thus, further studies are needed to investigate the neuroprotective effects of activation of TREM2 with apoE-mimic peptide after ICH by using the two models.

## Conclusions

In conclusion, we demonstrated that TREM2 activation with apoE-mimic peptide attenuated neuroinflammation and neuronal apoptosis after ICH, which was, at least in part, mediated by activation of PI3K/Akt signaling pathway. Therefore, TREM2 activation may be a potential therapeutic strategy in the management of ICH patients.

## Supplementary information

**Additional file 1: Table S1.** Summary of experimental groups and mortality rate in the study.

## Data Availability

The datasets analyzed during the current study are available from the corresponding author on reasonable request.

## Abbreviations

Akt: Protein kinase B
apoE: Apolipoprotein E
BBB: Blood-brain barrier
CNS: Central nervous system
DMSO: Dimethylsulfoxide
FJC: Fluoro-Jade C
ICH: Intracerebral hemorrhage
IL: Interleukin
PBS: Phosphate-buffered saline
PI3K: Phosphatidylinositol 3-kinase
siRNA: Small interfering RNA
TNF: Tumor necrosis factor
TREM2: Triggering receptor expressed on myeloid cells 2
TUNEL: Terminal deoxynucleotidyl transferase dUTP nick end labeling
