# Supplementary material for: TREM2 activation attenuates neuroinflammation and neuronal apoptosis via PI3K/Akt pathway after intracerebral hemorrhage in mice
Source: J Neuroinflammation. 2020 May 28;17:168. doi: 10.1186/s12974-020-01853-x (PMC7257134; doi:10.1186/s12974-020-01853-x)
Supplement: Supplementary file 1 — Additional file 1: Table S1. Summary of experimental groups and mortality rate in the study. [file 12974_2020_1853_MOESM1_ESM.docx]

| Table S1. Summary of experimental groups and mortality rate in the study. | | | | | | |
| --- | --- | --- | --- | --- | --- | --- |
|  |  |  |  |  |  |  |
| **Experimental Groups** | **Neurological test** | **IHC** | **WB** | **Exclusion** | **Mortality (%)** | **Subtotal** |
| **Experimental 1** |  |  |  |  |  |  |
| Sham |  |  | 6 | 0 | 0 | 6 |
| ICH (3h, 6h, 12h, 24h, 72h) |  | 2 | 30 | 0 | 3(8.57%) | 35 |
| **Experimental 2** |  |  |  |  |  |  |
| Sham | 12 |  |  | 0 | 0 | 12 |
| ICH + Vehicle | 12 |  |  | 0 | 2(14.29%) | 14 |
| ICH + COG1410 70μg/kg | 6 |  |  | 1 | 1(12.50%) | 8 |
| ICH + COG1410 200μg/kg | 12 |  |  | 0 | 1(7.69%) | 13 |
| ICH + COG1410 600μg/kg | 6 |  |  | 0 | 1(14.29%) | 7 |
| **Experimental 3** |  |  |  |  |  |  |
| Sham |  | 6 | 6 | 0 | 0 | 12 |
| ICH + Vehicle |  | 6 | 6 | 1 | 1(7.14%) | 14 |
| ICH + COG1410 |  | 6 | 6 | 0 | 1(7.69%) | 13 |
| **Experimental 4** |  |  |  |  |  |  |
| Sham | 8 |  | 0 | 0 | 0 | 8 |
| ICH + Vehicle | 8 |  | 0 | 0 | 1(11.11%) | 9 |
| ICH + COG1410 | 8 |  | 0 | 1 | 1(10.00%) | 10 |
| **Experimental 5** |  |  |  |  |  |  |
| Naïve + scr siRNA |  |  | 6 | 0 | 0 | 6 |
| Naïve + TREM2 siRNA |  |  | 6 | 0 | 0 | 6 |
| ICH + scr siRNA |  |  | 6 | 0 | 1(14.29%) | 7 |
| ICH + TREM2 siRNA |  |  | 6 | 0 | 1(14.29%) | 7 |
| ICH + COG1410 + TREM2 siRNA |  |  | 6 | 0 | 2(25.00%) | 8 |
| ICH + COG1410 + scr siRNA |  |  | 6 | 0 | 1(14.29%) | 7 |
| ICH + COG1410 + LY294002 |  |  | 6 | 1 | 1(12.50%) | 8 |
| ICH + COG1410 + DMSO |  |  | 6 | 0 | 0 | 6 |
| **Total** | 72 | 20 | 102 | 4 | 18(10.84%) | 216 |
| ICH, intracerebral hemorrhage; WB, western blot; IHC, immunohistochemistry; DMSO, dimethyl sulfoxide | | | | | | |
